# Methodological Tutorial Series for Epidemiological Studies: Confounder Selection and Sensitivity Analyses to Unmeasured Confounding From Epidemiological and Statistical Perspectives

**DOI:** 10.2188/jea.JE20240082

**Published:** 2025-01-05

**Authors:** Kosuke Inoue, Kentaro Sakamaki, Sho Komukai, Yuri Ito, Atsushi Goto, Tomohiro Shinozaki

**Affiliations:** 1 Department of Social Epidemiology, Graduate School of Medicine, Kyoto University, Kyoto, Japan; 2 Hakubi Center for Advanced Research, Kyoto University, Kyoto, Japan; 3 Center for Data Science, Yokohama City University, Yokohama, Japan; 4 Division of Biomedical Statistics, Department of Integrated Medicine, Graduate School of Medicine, Osaka University, Osaka, Japan; 5 Department of Medical Statistics, Research & Development Center, Osaka Medical and Pharmaceutical University, Osaka, Japan; 6 Department of Public Health, School of Medicine, Yokohama City University, Yokohama, Japan; 7 Department of Information and Computer Technology, Faculty of Engineering, Tokyo University of Science, Tokyo, Japan

**Keywords:** confounder, confounding, DAG, sensitivity analysis

## Abstract

In observational studies, identifying and adjusting for a sufficient set of confounders is crucial for accurately estimating the causal effect of the exposure on the outcome. Even in studies with large sample sizes, which typically benefit from small variances in estimates, there is a risk of producing estimates that are precisely inaccurate if the study suffers from systematic errors or biases, including confounding bias. To date, several approaches have been developed for selecting confounders. In this article, we first summarize the epidemiological and statistical approaches to identifying a sufficient set of confounders. Particularly, we introduce the modified disjunctive cause criterion as one of the most useful approaches, which involves controlling for any pre-exposure covariate that affects the exposure, outcome, or both. It then excludes instrumental variables but includes proxies for the shared common cause of exposure and outcome. Statistical confounder selection is also useful when dealing with a large number of covariates, even in studies with small sample sizes. After introducing several approaches, we discuss some pitfalls and considerations in confounder selection, such as the adjustment for instrumental variables, intermediate variables, and baseline outcome variables. Lastly, as it is often difficult to comprehensively measure key confounders, we introduce two statistics, *E-value* and *robustness value*, for assessing sensitivity to unmeasured confounders. Illustrated examples are provided using the National Health and Nutritional Examination Survey Epidemiologic Follow-up Study. Integrating these principles and approaches will enhance our understanding of confounder selection and facilitate better reporting and interpretation of future epidemiological studies.

## INTRODUCTION

Identifying and adjusting for a sufficient set of variables to estimate the causal effect of exposure on outcomes in observational studies has been a long-standing challenge for epidemiologists.^1^ Due to the rapid increase in available large datasets and the ethical challenges of conducting randomized trials, observational studies have played an important role in health science research. Although a large sample size often provides the benefit of having a small variance in the estimates, they also have a risk of producing estimates that are precisely inaccurate if the studies suffer from systematic errors or biases including confounding bias.^2^^,^^3^ Confounding is one of the major types of systematic biases and introduces bias in effect estimates when we do not measure or control for variables that can explain part or all of the observed association between the exposure and the outcome.^1^ Such variables are called confounders, and searching for a sufficient set of confounders is the most basic and best practice to avoid potential confounding biases in observational studies.

Several approaches have been proposed to identify such a sufficient set of confounders.^4^ One example is the use of causal diagrams, where complex causal relationships among variables are depicted using nodes and arrows.^5^^,^^6^ Meanwhile, understanding and accurately representing the true causal structure related to a research question can often be challenging. Another example is a statistical approach, including machine learning algorithms.^7^ However, these techniques cannot always confirm the temporal ordering of variables or distinguish confounders from mediators or colliders (variables affected by exposure) without prior knowledge. Given that researchers must apply these approaches on a case-by-case basis in their research—considering factors such as the research question, data availability, and existing evidence—a paper comprehensively covering both epidemiological and statistical perspectives on this topic is needed.

In this article, we first describe the principles of confounder selection from both an epidemiological and a statistical perspective. Subsequently, we explore some pitfalls of confounder selection, followed by an illustrative example. We also introduced two common approaches, *E-value*^8^ and *robustness value*,^9^ to assess the sensitivity to unmeasured confounders because it is often difficult to comprehensively measure confounders in the observational setting. Integrating these principles and approaches will enhance our understanding in confounder selection and facilitate better reporting and interpretation of epidemiological studies.

## CONFOUNDER SELECTION FROM EPIDEMIOLOGICAL PERSPECTIVES

**Figure 1.**
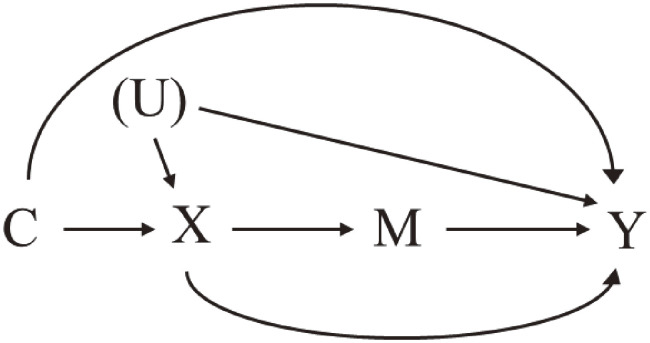
Notations of directed acyclic graph in this tutorial U with parenthesis, unmeasured variables that affect both confounders and outcomes. Note that a directed acyclic graph must include a common cause of any two variables already on the graph to be “causal,” even though we do not have information on that variable in our data.

In this section, we describe our approach to using causal diagrams, represented as directed acyclic graphs (DAGs), for confounder selection. These DAGs are instrumental in illustrating causal relationships between variables (ie, nodes), each represented by an arrow depicting the effect of one variable on another. It is important to note that in DAGs, a variable cannot be the cause of itself, hence the term ‘acyclic’. As shown in Figure 1, *X* represents the exposure, *Y* the outcome, *M* the mediator, and *C* a set of confounders. To illustrate, the arrow *X*→*Y* indicates the direct effect of *X* on *Y*.

There are two main types of paths in DAGs: open and closed. One specific type of closed path is the collider path which includes at least one node that receives two or more arrows—for example, a variable affected by both exposure and outcome. This node is called a collider. Although the collider paths do not induce statistical association as they are closed, conditioning on colliders or their effects can open the paths which induce the non-causal association that would lead to bias called *collider-stratification bias* (an example is shown below).

A causal DAG is a useful tool for a comprehensive identification of variables that address all confounding paths, and simultaneously precludes unnecessary overadjustment. Theoretically, the “backdoor” criterion is a gold-standard approach to find a sufficient set of confounders within whose strata the causal “exchangeability” between exposure groups holds^5^ by “blocking” all open backdoor paths (ie, open paths that start with an arrow pointing to the exposure) between *X* and *Y* in DAGs. However, we often do not have detailed information on causal structure across covariates, which limits us in applying the backdoor criterion. Herein, we review some practical approaches to find a sufficient set of confounders in epidemiological studies.^4^

### Criterion based on chronological order

**Figure 2.**
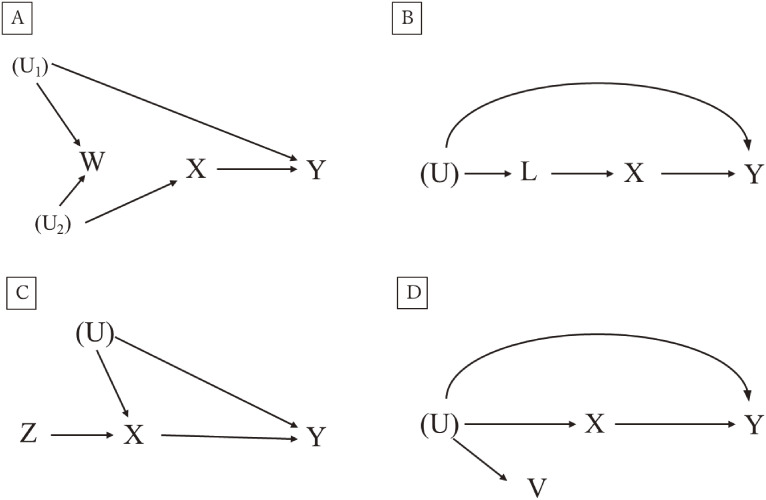
Directed acyclic graphs illustrating several scenarios for confounder selection principles U with parenthesis, unmeasured variables that affect both confounders and outcomes.

The “pretreatment (or preexposure) criterion” is a simple principle in the selection of covariates for confounding control.^10^ In this criterion, we control for all variables preceding treatment or exposure, since adjusting for covariates affected by the exposure has a risk of blocking the specific effects. The rationale lies in the understanding that any shared cause of both exposure and outcome is inherently pre-exposure. Due to uncertainties in how certain covariates impact both exposure and outcome, adjusting for all pre-exposure covariates based on this criterion seems to be a reasonable approach. However, this “pre-treatment” method has a risk of introducing *collider-stratification bias* or *M-bias* because it may select variable *W*, which is not a confounder despite the non-null association with both *X* and *Y*, as shown in Figure 2A. This is one of the “overadjustment” situations (ie, adjustment causes additional bias in the estimate).

### Criteria based on (partly postulated) causal structures

The “common cause criterion” is an alternative approach for confounder selection, which circumvents the aforementioned *M-bias*. This approach adjusts for all of the pre-treatment causes shared by exposure and outcome.^11^ However, this approach also has a limitation when data on certain shared causes are unavailable. In such cases, other covariates, not identified by this criterion, might still adequately control for confounding. (ie, *L* in Figure 2B).

An alternative approach “disjunctive cause criterion” balances these existing approaches (ie, pretreatment criterion and common cause criterion) by controlling for any pre-exposure covariate affecting the exposure, outcome, or both.^12^ This approach avoids including *W* in Figure 2A and ensures controlling for *L* in Figure 2B, thus overcoming the limitations of “pre-treatment criterion” and “common cause criterion”.

When addressing confounding control in epidemiological studies, the disjunctive cause criterion offers a systematic approach, especially when the causal relationship of covariates with exposure and outcome is known. Its theoretical promise, however, comes with nuances for practical application. Specifically, controlling for a variable that solely affects the exposure can inadvertently amplify bias due to an unmeasured covariate, a phenomenon known as the *Z-bias*. These specific variables, referred to as *instrumental variables* (IVs), while advantageous in certain analytic frameworks, can introduce more bias when they are controlled for by conditioning in regression or propensity score models (Figure 2C). Thus, it is additionally needed to exclude these IVs when implementing the disjunctive cause criterion. More details in *Z-bias* will be discussed below. Furthermore, controlling proxies *V* for variables *U* that affect both exposure and outcome is also helpful to close the back-door path, particularly when *U* is not measured (Figure 2D). Modified version of the disjunctive cause criterion—“modified disjunctive cause criterion”—with these two additional rules (ie, removing IVs and including proxies for shared common cause of exposure and outcome) is considered to be one of the most comprehensive approaches to identify a sufficient set of confounders in the partly specified causal DAGs.^4^

## CONFOUNDER SELECTION FROM STATISTICAL PERSPECTIVES

When the number of covariates is large compared to the sample size, sparse-data bias and imprecise estimates (ie, large estimated standard errors) may become practical concerns.^13^ Moreover, the high-dimensional covariates make it a daunting task to specify regression models with at least approximately correct form.^14^ Hence, we may need to reduce the number of covariates to avoid these problems, even after the above-mentioned epidemiological confounder selection procedure has been used. In this section, we introduce several approaches to select confounders from statistical perspectives in regression models.

### Backward and forward selections

Historically, backward and forward selection have been commonly used in statistical covariate (not necessarily confounders) selection.^15^ Covariates are usually selected by considering the relationship with the exposure and the outcome. Backward selection starts with the full set of covariates, methodically discarding each one not strongly associated with the outcome conditional on exposure and other covariates. Instead, forward selection starts with a minimum set of covariates, progressively introducing those associated with outcomes conditional on exposure and those already selected. In both approaches, variable selections are based on *P*-values of coefficients, goodness of model fit (eg, Akaike Information Criterion, Bayesian Information Criterion, and Mallows’ C_p_), and *change in estimate*, described below. For example, when variables are selected based on *P*-values, the cutoff value is commonly set at 0.05, but it can vary across studies. Although both methods are intuitively appealing, they encounter several practical challenges. For example, covariates chosen from these statistical procedures are not necessarily confounders. In addition, once covariates are chosen, fitting them into a final regression model does not always ensure valid estimates and confidence intervals (CIs) (ie, the problem of *selective inference*^16^).

### Change in estimates

The change-in-estimate approach provides one of the criteria to statistically select covariates in the model.^17^ Decisions in this approach are influenced by the degree to which a covariate alters the estimate of the causal effect of interest (eg, a regression coefficient of exposure), often by a threshold, such as 10%. While it evaluates empirical associations similarly to the above methods, it diverges by focusing directly on the magnitude of effect estimates of exposure instead of the presence of an association or a *P*-value threshold for outcome-covariate relationship. It is also imperative that the initial set of covariates sufficiently controls for confounding; using a small set of covariates without this assurance may inadvertently introduce bias.^7^^,^^17^ Limitations of this method include arbitrary setting of threshold and its dependence on effect measures; the obtained estimates may not be interpretable for non-collapsible measures, such as odds ratios or hazard ratios, which correspond to coefficients in logistic or Cox models, when outcomes are frequent.

### Penalization

Penalization is a type of shrinkage estimation method specifically designed for analyzing high-dimensional datasets where the number of variables exceeds the number of observations. By introducing penalty terms, this approach effectively addresses the limitations of traditional statistical methods in such scenarios, optimizing the balance between bias and variance.^18^ Lasso, ridge, and elastic net regression are popular penalization approaches that offer tailored solutions depending on the data structure and research objectives. Firth bias adjustment is another type of penalization designed to mitigate the bias inherent in the maximum likelihood estimates even when dealing with limit event numbers (ie, to correct the small sample bias of the maximum likelihood estimates).^19^ In logistic regression, where the log odds of the outcome is modeled as a linear combination of predictors, the standard likelihood of unknown parameter *β* (ie, *L*[*β*]) can sometimes produce biased coefficient estimates, especially with sparse data or complete separation. The Firth method introduces a penalty to this likelihood, resulting in a modified likelihood L*(*β*) = *L*(*β*) × |*I*(*β*)|^1/2^, where *I*(*β*) is the Fisher information matrix. By penalizing the likelihood function in this manner, the Firth adjustment yields more reliable and unbiased estimates, particularly crucial in epidemiological and biomedical research scenarios.

### High-dimensional propensity score

Confounder selection can also be implemented through propensity score modeling. The high-dimensional propensity score (HDPS) is one such approach in statistical covariate selection.^20^^,^^21^ In brief, HDPS select covariates in order of the approximate estimate of the bias each covariate may generate using the following seven steps.

1. Specify the data dimensions (eg, demographic characteristics, disease history, prescription, biomarkers).
2. Identify empirical candidate covariates (eg, the top *k* most prevalent covariates).
3. For the top *k* most prevalent covariate in each dimension identified in the previous step, the occurrence is assessed based on how frequently each covariate (eg, diagnosis code) was recorded.
4. Within each data dimension, assess potential biases for candidate variables. In this step, we compare the multiplicative bias term ( ${Bias}_{M}=\frac{P_{c_{1}}({RR}_{cd}-1)+1}{P_{c_{0}}({RR}_{cd}-1)+1}$ , where $P_{c_{1}}$ and $P_{c_{0}}$ are the prevalences of a binary confounding factor in the exposed and unexposed groups, respectively, and *RR_cd_* is the independent association between a confounder and the outcome) across each covariate.
5. From the set of prioritized covariates based on the bias term (ie, top *k* covariates in descending order of |log(*Bias_M_*)|), select covariates that will be included in the propensity score model.
6. Estimate propensity score using multivariable logistic regression using the selected covariates and additional covariates based on prior knowledge.
7. Lastly, the exposure-outcome association is estimated using the propensity score analysis (eg, covariate adjustment, matching, inverse probability of weighting, and stratification).

More recently, the outcome-adaptive lasso approach has also been used to select covariates based on the information on the covariate-outcome relationships (rather than covariate-exposure relationships) in the high-dimensional settings.^22^ This approach allows us to exclude covariates associated with the outcome only through the exposure (ie, IV) when selecting confounders, resulting in increased statistical efficiency with unbiased treatment effect estimation. Lastly, it is important to note that obtaining unbiased effect estimates may require not only appropriate variable selection but also a nearly correct model specification. An option could be to use flexible nonparametric estimation methods, such as targeted learning with Super Learner,^23^ following the variable selection to achieve approximately unbiased effect estimates. However, such flexibility might lead to variance inflation and finite-sample bias, particularly in relatively small datasets that necessitate variable selection procedures. With parametric regression models, although the “adaptive” Lasso has been reported to be robust against model misspecification concerning the selection of outcome predictors,^24^ it remains uncertain whether the resulting estimates are unbiased for causal effects. Since there are no one-size-fits-all strategies in scenarios that require both variable selection and model specification, conducting sensitivity and bias analyses could be beneficial, as demonstrated in the data analysis section.

## PITFALLS OF CONFOUNDERS SELECTION

When conducting epidemiologic studies, we often encounter pitfalls in selecting confounders. In this section, we describe three of such key pitfalls.

### Adjusting for instrumental variables

In point-treatment settings, as described in the “modified disjunctive cause criterion”, conditioning on IVs that are associated solely with the outcome through the treatment can increase bias in treatment effect estimates, especially when unmeasured confounding exists (Figure 2C).^25^^,^^26^ This bias also extends to near-IVs, variables only weakly tied to the outcome, not through the treatment. These variables have been termed ‘bias amplifiers’ in prior research.^25^^,^^26^ Studies have emphasized the inherent challenges in differentiating between genuine confounders and IVs, and thus underscored the importance of carefully including dominant predictors of the treatment when constructing models. A more recent study using g-computation algorithm demonstrated that adjusting for an IV can further exacerbate this bias in the time-varying settings as well.^27^ This bias amplification was observed even when adjusting for near-IV when such a variable had a very weak association with unmeasured exposure-outcome confounders.

### Adjusting for mediators

**Figure 3.**
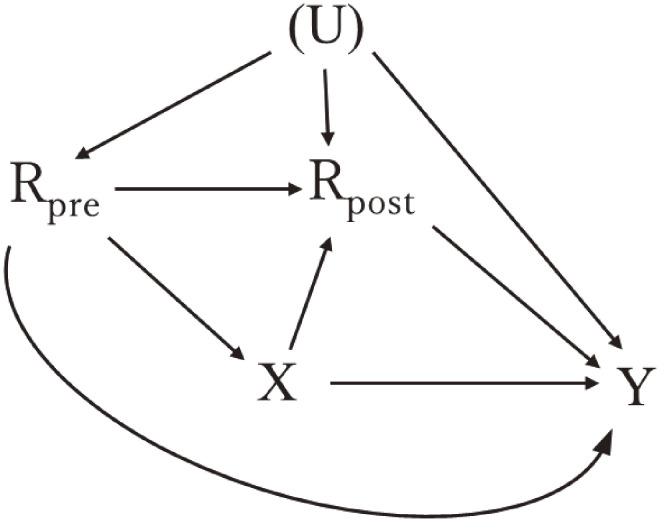
Directed acyclic graphs illustrating time-varying covariates measured before and after the exposure U with parenthesis, unmeasured variables that affect both confounders and outcomes. *R_pre_* and *R_post_* showed measured covariates *R* measured before (*R_pre_*) and after (*R_post_*) the exposure.

When the exposure and covariates are measured at the same timing, it is often difficult to clarify whether variables serve as confounders or mediators, so-called *confounder-mediator dilemma*,^28^ because the temporal sequence of study variables within a dataset remains ambiguous (Figure 3). For example, many epidemiological studies examining the link between physical activity and health outcomes provide obesity data only at the start, making it hard to differentiate between confounders (*R_pre_* in Figure 3) and mediators (*R_post_* in Figure 3) due to the lack of a clear time ordering. Even when studies are longitudinal, it is challenging to determine if obesity measurements reflect status before or after physical activity, as both represent accumulations from previous years. In such a scenario, it is sometimes recommended to compare and report both results with and without adjustment of obesity.

### Adjusting for baseline outcome variables

In observational studies, there is also a controversy about whether baseline outcome variables should be included in the model or not. A traditional example is the relationship between education and changes in cognition.^29^ When “baseline” cognitive function is affected by educational status, incorporating these baseline values can inadvertently introduce a *collider-stratification bias* for education effects on cognitive trajectory, which corresponds to the bias due to conditioning on *R_post_* in the collider path *X*→*R_post_*←(*U*)→*Y* in Figure 3. On the other hand, when “baseline” cognitive function was measured before obtaining education (so, life-course follow-up is needed), analysts would be better off adjusting for such early-life variables because they may explain unmeasured cognitive differences between individuals (ie, conditioning on *R_pre_* is needed to close open backdoor paths *X*←*R_pre_*→*Y*, *X*←*R_pre_*←[*U*]→*Y*, *X*←*R_pre_*→*R_post_*→*Y*, and *X*←*R_pre_*←[*U*]→*R_post_*→*Y* in Figure 3). Because the term “baseline” is used in different ways in observational studies, the causal structure incorporating chronological order with causal DAGs would be helpful in deciding whether the baseline outcome variable should be adjusted for.

## SENSITIVITY ANALYSIS FOR UNMEASURED CONFOUNDERS

Although it is important to understand how to select a sufficient set of confounders, it is often difficult to measure all these variables. In such a scenario, sensitivity analyses are helpful to interpret the robustness of the results to unmeasured confounders. Here, we introduce two common statistics: *E-value* and *robustness value*.

### E-value

In 2016, VanderWeele and Ding recently introduced the *E-value*, a metric quantifying the minimum strength of associations among unmeasured confounders, exposure, and outcome to thoroughly account for an observed association, given the measured covariates.^8^^,^^30^ Following a risk ratio (RR), the *E-value* is estimated as $RR+\sqrt{RR\times (RR-1)}$ , with RR denoting the observed risk ratio after considering measured confounders. When RR is less than 1, we need to take the inverse of the observed RR and then apply the formula. In cases of an odds ratio (OR) or a hazard ratio (HR), one can substitute RR with OR or HR for infrequent outcomes (<15%). For prevalent outcomes (≥15%), the *E-value* for the OR can be obtained by approximating RR by taking the square root of the OR, and the *E-value* for the HR can be obtained by approximating RR by calculating (1 − 0.5^sqrt(HR)^)/(1 − 0.5^sqrt(1/HR)^).^31^ The *E-value* for risk difference (RD) can be obtained by replacing RR with the adjusted risk for the exposed group divided by the adjusted risk for the unexposed group in the formula. For continuous exposures, the *E-value* can be computed using the same increment used in assessing the association between the exposure and outcome.

### Robustness value

In 2020, Cinelli and Hazlett developed another approach to communicate the sensitivity of regression results to unmeasured confounding bias without assumptions on the treatment assignment mechanisms nor the distribution of the unmeasured confounders.^9^ In this approach, we calculate the *robustness value* that describes the association unobserved confounding would need to have with both the treatment and the outcome to explain away the observed association (in the absolute scale; ie, RD). The *robustness value* has a similar concept to *E-value* but is a measurement in terms of the percentage of variance explained instead of RR.^8^^,^^30^ The partial *R*^2^ of the treatment with the outcome can also be calculated to assess how robust the point estimate is to unmeasured confounders in an extreme scenario where confounders explain all residual variance of the outcome. Then, it formally bound the strength of unmeasured confounders as strong as certain measured covariate(s) in terms of the explained variance of the treatment and/or the outcome, which could be described in intuitive graphical tools as shown in the example illustration below.

## EXAMPLE ILLUSTRATION

### Settings

**Figure 4.**
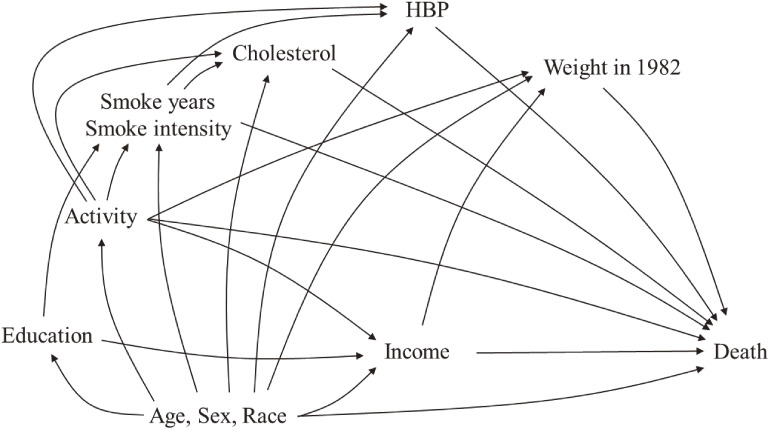
Directed acyclic graphs under the example illustration using NHEFS data All variables (except weight in 1982 and death) were collected in 1971. HBP, High blood pressure; NHEFS, the National Health and Nutritional Examination Survey Epidemiologic Follow-up Study.

In this example illustration, we analyzed data from 1,461 participants of the National Health and Nutritional Examination Survey (NHANES) Epidemiologic Follow-up Study (NHEFS).^32^ NHEFS, a national longitudinal study, was designed to investigate the relationships between clinical, nutritional, and behavioral factors identified in the original NHANES I study and subsequent morbidity, mortality, and hospital utilization. Initially, the sample design of NHEFS was a multistage, stratified, probability sample targeting the United States civilian non-institutionalized adult population, with an oversampling of specific groups like the elderly, women in their reproductive years, minorities, and low-income individuals. NHEFS followed the cohort of NHANES I participants aged 25 to 74 years from 1971 to 1975 during the baseline assessment. Data collection extended to follow-up visits in 1982–1984, 1986, 1987, and 1992. Our primary aim was to assess the relationship between low income (<$1,000 or not in 1971) and mortality until 1992. The outcome variable was binary: 0 for absence and 1 for presence of death until 1992. Covariates incorporated in our study included age in 1971, sex, race, education, smoking status (year of smoking; number of cigarettes smoked per day in 1971 [smoking intensity]), physical activity in 1971, total cholesterol levels (mg/100 mL) in 1971, high blood pressure (HBP) in 1971, and body weight in 1982. We hypothesize that Figure 4 illustrates the true causal relationship among these covariates. To simplify the analysis and focus on confounder selection in our example illustration, we excluded individuals with missing data on covariates (ie, complete-case analysis) under the assumption of missing completely at random, which is generally required to estimate marginal association measures unbiasedly.^33^

### Statistical analyses

We calculated RDs, RRs, and ORs with 95% CIs utilizing the regression standardization and the inverse probability weighting method using propensity scores. Multivariate logistic regression models were applied to build the model for the outcome and the model for the propensity score, respectively. Covariates for the models were chosen through both an epidemiological perspective (ie, pretreatment criterion, common cause criterion, modified disjunctive cause criterion, and backdoor criterion) and a statistical perspective (ie, backward elimination for the outcome model and the propensity score model; Table 1). The 95% CIs were estimated using both the theoretical methods and the bootstrap methods. In the bootstrap method, when variables were selected from a statistical perspective, confidence intervals were constructed by estimating the causal effect following variable selection in each of the 1,000 bootstrap samples. To assess the robustness of our findings, *E-value* and *robustness value* were calculated for the estimates in the models including covariates selected using the backdoor criterion.

**Table 1.** Summary of the selected covariates in each criterion of epidemiological perspective and statistical perspective

Criteria	The number of selected variables	Selected variables
Pretreatment criterion	9	Age, Sex, Race, Education, Smoke intensity, Smoke years, Physical activity, Cholesterol, High blood pressure
Common cause criterion	4	Age, Sex, Race, Physical activity
Modified disjunctive cause criterion	9	Age, Sex, Race, Education, Smoke intensity, Smoke years, Physical activity, Cholesterol, High blood pressure

Backward selection		
Outcome	7	Age, Sex, Smoke intensity, Smoke years, Physical activity, Cholesterol, High blood pressure
Propensity score	7	Age, Sex, Race, Education, Smoke intensity, Physical activity, Cholesterol
Disjunctive^a^	9	Age, Sex, Race, Education, Smoke years, Smoke intensity, Physical activity, Cholesterol, High blood pressure

Backdoor criterion	5	Age, Sex, Race, Education^b^, Physical activity

^a^The disjunctive in backward selection indicated controlling for the covariates selected at least one of the outcome model and propensity score model.

^b^In the backdoor criterion, smoke years and smoke intensity can also be selected instead of (or in addition to) education to close all back-door paths.

### Results

The RDs, RRs, and ORs for each model, derived from each confounder selection approach, are presented in Table 2. In this example, the pretreatment criterion, disjunctive cause criterion, and disjunctive backward selection criterion (which control for variables selected in at least one of either the outcome or the propensity score models) selected the same set of covariates as confounders. Based on the DAGs illustrated in Figure 4, these criteria, along with backward selection for propensity scores and the backdoor criterion, provided models that could appropriately eliminate the effects of confounding. On the other hand, the common cause criterion and backward elimination in the outcome regression model failed to address confounding due to some unblocked backdoor paths. Consistent estimates were obtained from criteria that were adjusted correctly (ie, backdoor criterion, pretreatment criterion, disjunctive cause criterion, backward elimination for propensity score, and disjunctive by backward elimination). In contrast, estimates derived using the common cause criterion and backward elimination for the outcome model diverged from those obtained through the backdoor criterion. From a statistical standpoint, while the standard construction of confidence intervals does not consider the uncertainty associated with variable selection, incorporating variable selection through the bootstrap method allows for the construction of a more reliable confidence interval.

**Table 2.** Summary of results estimated by the regression standardization and the inverse probability weighting method by propensity scores across each criterion

Estimand	Criterion	Regression standardization			IPTW		
		Estimate	95% CI	Bootstrap 95% CI	Estimate	95% CI	Bootstrap 95% CI
RD	Backdoor	0.044	[0.005–0.083]	[0.004–0.083]	0.062	[0.017–0.106]	[0.020–0.103]
	Pretreatment	0.050	[0.011–0.089]	[0.009–0.091]	0.066	[0.021–0.110]	[0.024–0.104]
	Common cause	0.063	[0.026–0.100]	[0.029–0.101]	0.079	[0.039–0.120]	[0.043–0.115]
	Disjunctive cause	0.050	[0.011–0.089]	[0.011–0.088]	0.066	[0.021–0.110]	[0.023–0.103]
	Backward (Outcome)	0.069	[0.032–0.105]	[0.014–0.093]			
	Backward (PS)				0.065	[0.020–0.109]	[0.026–0.103]
	Backward (Disjunctive)	0.050	[0.011–0.089]	[0.011–0.092]	0.066	[0.021–0.110]	[0.028–0.104]

RR	Backdoor	1.273	[1.026–1.579]	[1.019–1.569]	1.426	[1.097–1.855]	[1.113–1.838]
	Pretreatment	1.318	[1.061–1.638]	[1.045–1.674]	1.457	[1.120–1.894]	[1.152–1.863]
	Common cause	1.411	[1.149–1.733]	[1.177–1.739]	1.563	[1.236–1.976]	[1.267–1.911]
	Disjunctive cause	1.318	[1.061–1.638]	[1.062–1.628]	1.457	[1.120–1.894]	[1.136–1.855]
	Backward (Outcome)	1.460	[1.191–1.790]	[1.081–1.700]			
	Backward (PS)				1.448	[1.113–1.884]	[1.156–1.816]
	Backward (Disjunctive)	1.318	[1.061–1.638]	[1.063–1.647]	1.457	[1.120–1.894]	[1.160–1.860]

OR	Backdoor	1.343	[1.033–1.747]	[1.023–1.739]	1.538	[1.122–2.109]	[1.143–2.081]
	Pretreatment	1.402	[1.075–1.827]	[1.057–1.872]	1.577	[1.150–2.163]	[1.189–2.113]
	Common cause	1.524	[1.186–1.957]	[1.217–1.958]	1.721	[1.297–2.286]	[1.337–2.187]
	Disjunctive cause	1.402	[1.075–1.827]	[1.077–1.808]	1.577	[1.150–2.163]	[1.168–2.082]
	Backward (Outcome)	1.588	[1.239–2.035]	[1.100–1.902]			
	Backward (PS)				1.567	[1.142–2.149]	[1.191–2.047]
	Backward (Disjunctive)	1.402	[1.075–1.827]	[1.077–1.837]	1.577	[1.150–2.163]	[1.200–2.095]

CI, confidence interval; IPTW, inverse probability of treatment weighting; OR, odds ratio; PS, propensity score; RD, risk difference; RR, risk ratio.

Backward (Outcome) and Backward (PS) indicated the variable selection by backward elimination for the outcome regression model and the propensity score model, respectively, and Backward (Disjunctive) indicated controlling the variables selected at least one of the Backward (Outcome) and Backward (PS); the 95% CI indicated the 95% confidence intervals and the bootstrap 95% CI indicated the bootstrap confidence intervals.

### Sensitivity analyses

The *E-values* for the estimated RR (1.27; 95% CI, 1.03–1.58) using regression standardization, including covariates selected by backdoor criterion, was 1.86 (1.21 for lower 95% CI), meaning that an unmeasured confounder would need to be associated with both low income and 5-year mortality with an RR >1.86 conditional on measured covariates to fully explain away this observed association. Researchers then discuss whether such unmeasured confounders, associated with both exposure and outcome with a larger RR than the estimated *E-value*, are likely to exist or not to assess the robustness of their findings. Likewise, *E-values* were 1.58 for the estimated OR (1.34) and 1.46 for the estimated RD (+4.4 percentage point), respectively. Please note that these *E-values* were calculated for the prevalent outcome (18%) in our example dataset.

**Figure 5.**
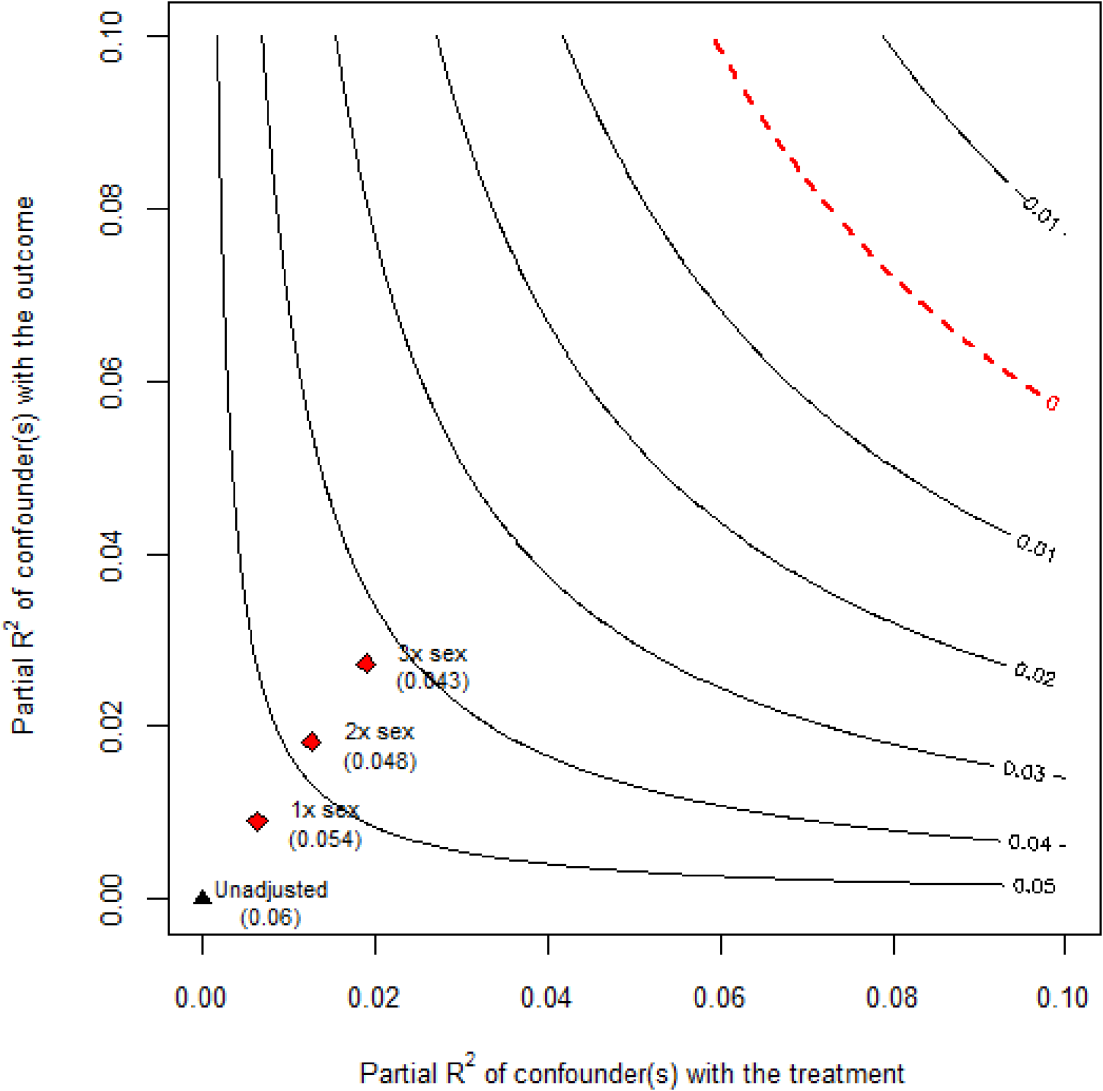
Sensitivity contour plot of the point estimate in the example illustration This plot shows that the association between income and mortality is robust to confounders even three times as strong as sex; sex was chosen based on domain knowledge and the strength of its association with both exposure and outcome. Black triangle (unadjusted) shows the estimated effect, and three red diamonds (1 × sex, 2 × sex, and 3 × sex) show what would be the estimate that one would have obtained in the regression model including unobserved confounders with such hypothetical strengths (ie, *n* times as strong as the observed sex).

The *robustness value* was calculated only for the estimates obtained from the ordinary least squares method, due to limitations in the current package availability.^34^ The *robustness value* of the estimated RD (+5.99 percentage point; 95% CI, 2.10–9.87) was 7.62% (2.73% for lower 95% CI), meaning that unmeasured confounders explaining at least 7.62% of the residual variance of both income and mortality would explain away the entire estimated effect. The partial *R*^2^ of the treatment with the outcome was 0.62%, meaning that if confounders explained 100% of the residual variance of the outcome (ie, an extreme scenario), they would need to explain at least 0.62% of the residual variance of the treatment to bring down the estimated effect to zero. The sensitivity plot revealed that the observed association between income and mortality is robust to confounders even three times as strong as sex (Figure 5). To understand the impact of unmeasured confounders relative to these metrics, we often use one of the most apparent confounders. In our case, we chose sex based on our domain knowledge and the strength of its association with both exposure and outcome.

## CONCLUSION

This tutorial paper summarizes the epidemiological and statistical approaches to identify a sufficient set of confounders in epidemiological studies. Modified disjunctive cause criterion is one of the useful approaches even without detailed information on causal structure across covariates. Confounder selection from statistical perspectives is also useful particularly when the number of covariates is large despite a small sample size. If some key covariates are unmeasured, researchers may want to consider sensitivity analyses, such as quantitative bias analyses, *E-value* calculations, and *robustness value* calculations. Epidemiologists are recommended to carefully select confounders based on these approaches and considerations when investigating the causal relationship between exposure and outcome.
